# Comparative proteomic analysis between nitrogen supplemented and starved conditions in *Magnaporthe oryzae*

**DOI:** 10.1186/s12953-017-0128-y

**Published:** 2017-11-13

**Authors:** Yeonyee Oh, Suzanne L. Robertson, Jennifer Parker, David C. Muddiman, Ralph A. Dean

**Affiliations:** 1 Center for Integrated Fungal Research, Department of Entomology and Plant Pathology, North Carolina State University, Raleigh, NC 27695 USA; 2 W. M. Keck FT-ICR Mass Spectrometry Laboratory, Department of Chemistry, North Carolina State University, Raleigh, NC 27695 USA

**Keywords:** Proteomics, *Magnaporthe oryzae*, Nitrogen starvation, Melanin biosynthesis, Extracellular protein

## Abstract

**Background:**

Fungi are constantly exposed to nitrogen limiting environments, and thus the efficient regulation of nitrogen metabolism is essential for their survival, growth, development and pathogenicity. To understand how the rice blast pathogen *Magnaporthe oryzae* copes with limited nitrogen availability, a global proteome analysis under nitrogen supplemented and nitrogen starved conditions was completed.

**Methods:**

*M. oryzae* strain 70–15 was cultivated in liquid minimal media and transferred to media with nitrate or without a nitrogen source. Proteins were isolated and subjected to unfractionated gel-free based liquid chromatography-tandem mass spectrometry (LC-MS/MS). The subcellular localization and function of the identified proteins were predicted using bioinformatics tools.

**Results:**

A total of 5498 *M. oryzae* proteins were identified. Comparative analysis of protein expression showed 363 proteins and 266 proteins significantly induced or uniquely expressed under nitrogen starved or nitrogen supplemented conditions, respectively. A functional analysis of differentially expressed proteins revealed that during nitrogen starvation nitrogen catabolite repression, melanin biosynthesis, protein degradation and protein translation pathways underwent extensive alterations. In addition, nitrogen starvation induced accumulation of various extracellular proteins including small extracellular proteins consistent with observations of a link between nitrogen starvation and the development of pathogenicity in *M. oryzae*.

**Conclusion:**

The results from this study provide a comprehensive understanding of fungal responses to nitrogen availability.

**Electronic supplementary material:**

The online version of this article (10.1186/s12953-017-0128-y) contains supplementary material, which is available to authorized users.

## Background

In nature, fungi, including plant pathogens, are often exposed to environments where nutrients are insufficient. To deal with such conditions, several survival mechanisms are activated such as intercellular nutrient recycling, the scavenging of resources and/ or morphological changes to aid growth and proliferation in stressful surroundings. On the plant surface, potential pathogenic fungi are often limited for nutrients until the host has been successfully infected. Indeed, the expression of pathogenicity genes are frequently elevated during nitrogen limiting conditions suggesting that nitrogen starvation is a driving force for successful fungal infection of their host organisms [1–3]. Unlike their plant hosts, fungi can utilize diverse compounds as nitrogen sources from primary preferred sources such as ammonium, glutamine and glutamate to other non-preferred secondary forms including nitrate, nitrite, other amino acids and proteins. Assimilation of nitrogen is tightly regulated by global regulators that ensure use of preferential nitrogen sources over less desirable sources [4]. AreA, Nit2 and NUT1, which encode GATA type transcription factors are well known as a key regulatory genes in *Aspergillus nidulans*, *Neurospora crassa* and *Magnaporthe oryzae* respectively [5–7]. A number of studies have linked nitrogen metabolism regulation with the ability to cause disease by plant pathogenic fungi, including *Cladosporium fulvum*, *Colletotrichum lindemuthianum* and *M. oryzae* [7–9].

The rice blast pathogen, *M. oryzae,* is the most significant fungal pathogen of rice crops worldwide as it routinely destroys rice production by 10–30% [10]. *M. oryzae* also infects other agronomically important grass species including wheat, barley and millet [11]. As typical of many filamentous ascomycete fungal pathogens, *M. oryzae* develops a specialized infection structure, the appressorium, to attach and penetrate the host plant. After successful colonization of host tissues, necrotic lesions form within a few days, from which conidia are produced rapidly spreading the disease to neighboring plants under favorable conditions [12].

Over the past few years, transcriptome and proteome studies during nitrogen starvation are beginning to reveal how nitrogen availability affects phytopathogens [13, 14]. In transcriptome studies of *M. oryzae*, 520 genes showed increased gene expression during nitrogen starvation, the majority of which were involved in amino acid metabolism and uptake [13]. We found the important pathogenicity factor, SPM1, a putative subtilisin-like protease was significantly induced under nitrogen starvation as well as during appressorium formation and in response to exogenous cyclic AMP treatment [15]. Further study showed that SPM1 was localized in vacuoles and involved in autophagy during appressorium formation. Infectious growth of the spm1 deletion mutant in rice epidermal cells was very limited [16]. In other phytopathogenic fungi such as in *Verticillum dahliae*, the expression of 487 genes, which included genes involved in melanin biosynthesis, were significantly upregulated under nitrogen starvation [14]. Furthermore, analyses of upregulated genes under nitrogen stress has yielded numerous small secreted proteins, a feature typical of many fungal effectors [17, 18].

Proteomic analysis of liquid media from *V. dahliae* culture grown under nitrogen starvation showed enrichment of proteins for cell wall degradation, reactive oxygen species (ROS) scavenging and stress response as well as protein and carbohydrate metabolism [18]. Up to now, studies of the *M. oryzae* proteome under nitrogen starvation have been conducted through analysis of two-dimensional gels coupled with mass spectrometry analysis. An analysis of liquid culture media identified 85 putative secreted proteins upregulated under nitrogen starvation. The majority were cell wall hydrolase enzymes, protein and lipid hydrolases and proteins for ROS detoxification [17]. Another study reported 975 protein spots from complete media and 1169 spots under nitrogen limitation conditions. Forty three differentially accumulated proteins were identified, of which several were found to be involved in glycolysis, the tricarboxylic acid cycle and nitrogen metabolism [19].

In this comprehensive study, unfractionated protein samples coupled with advanced mass spectrometry technology was employed to identify and monitor more than 40% of the predicted *M. oryzae* entire proteome during nitrogen starvation, which revealed key biological information pertaining to its survival and pathogenicity.

## Methods

### Materials

All reagents were purchased from Sigma-Aldrich (St. Louis, MO) and all solvents were HPLC-grade from Honeywell Burdick & Jackson (Muskegon, MI), unless otherwise stated.

### Fungal growth and protein extraction

*M. oryzae* strain 70–15 conidia were harvested from 1 week old V8 agar plates and inoculated into complete liquid medium (10 g sucrose; 1 ml of 1000X trace elements (2.2 g ZnSO_4_, 1.1 g H_3_BO_3_, 0.5 g MnCl_2_-4H_2_O, 0.5 g FeSO_4_-7H_2_O, 0.17 g CoCl_2_, 0.16 g CuSO_4_-5H_2_O, 0.15 g Na_2_MoO_4_-2H_2_O and 5 g disodium EDTA per 100 ml); 6 g casein acid hydrolysate; 6 g yeast extract in 1 L). The culture was grown at 28 °C on a 200 rpm shaker for 3 days. The mycelial mat was then collected on sterile filter paper, washed three times with sterile distilled water, and divided into six equal pieces. Biological replicates were grown by placing one piece of mycelial mat into each of the three flasks containing minimal media supplemented with nitrogen (N+), or three flasks containing nitrogen- limiting (N-) media. N+ media contained 10 g sucrose, 1 ml of 1000X trace element solution, 50 ml nitrate salts (60 g NaNO_3_, 5.2 g KCl, 5.2 g MgSO_4_.∙ 7H_2_O, and 15.2 g KH_2_PO_4_ for 500 ml), 1 mg thiamine and 5 μg biotin per liter. N- media was the same as N+ except lacked NaNO_3_. The pH of the media was adjusted to 6.5 with NaOH. These cultures were grown at 28 °C in a 200 rpm shaker. After 12 h, the mycelial mats were collected, washed with sterile distilled water and then ground into powder with liquid nitrogen. Proteins were extracted using lysis buffer (50 mM HEPES (pH 7.5), 0.5% Nonidet P-40, 250 mM NaCl, 10% (*v*/v) glycerol, 2 mM EDTA (pH 8.0), one cOmplete™ ULTRA tablet Protease Inhibitor cocktail (Roche, Germany) per 50 ml). Lysate was clarified by centrifugation at 16,000 g for 15 min. Protein concentration was estimated using the Pierce™ Coomassie Bradford assay kit (Thermo Fisher Scientific, Waltham, MA). Samples were stored at −80 C.

### Sample preparation and digestion

Filter aided sample preparation was performed as in Loziuk et al. [20] with slight modifications. For each biological replicate, a volume of lysate containing 250μg of protein was reduced in 50 mM Dithiothreitol (DTT) in 8 M urea and 50 mM tris-HCl (denaturing/alkylating buffer) at 56 °C for 30 min. Cysteine residues were alkylated in the dark with 10 mM N-ethylmaleimide (NEM) for 30 min at room temperature. Samples were transferred to a 10 kDa molecular weight cutoff centrifugation filter (EMD Millipore, Billerica, MA) and centrifuged at 14000 g for 15 min at 20 °C (all centrifugation steps performed with these settings). Buffer exchange with 2 M urea, 10 mM calcium chloride was performed three times by centrifugation. Samples were digested in the filter at 37 °C for 12 h using a 1:50 modified porcine trypsin to sample ratio. Peptides were eluted by centrifugation, and quenched with 1% formic acid, 0.001% Zwittergent 3–16 (Calbiochem, La Jolla, CA) for further analysis. The concentration of peptides was measured by the NanoDrop™ (Thermo Scientific).

### LC-MS/MS

Reverse-phase nano-LC was performed with an EASY nLC 1000 (Thermo Fisher, Waltham, MA) using a 20 cm, 75 um I.D Picofrit column (New Objective, Woburn, MA) packed with Kinetix 2.6 μm (100 Å) stationary phase (Phenomenex, Torrance, CA). One microgram of peptides was loaded onto the column and eluted at a flow rate of 300 nL/min with a 240 min linear gradient (5–30%). Buffers consisted of mobile phase A (98% H_2_O, 2% ACN, and 0.2% FA) and mobile phase B (2% H_2_O, 98% ACN, and 0.2% FA). Three technical replicates were performed per sample, and data was collected on a Q-Exactive High Field mass spectrometer (Thermo Fisher Scientific, Waltham, MA). For MS^1^ scans, resolving power was 120,000, the AGC target was 3e6, an injection time of 50 ms was applied, and the scan range was set to 300–1600 m/z. During top-20 data dependent MS^2^ scans, resolution was 15,000, the AGC was 1e5, an injection time of 30 ms was applied, the scan range was 200–2000 m/z, a 2 m/z isolation window was used, normalized collision energy was set to 27, the underfill ratio was 2% with an intensity threshold of 6.7e4, and a dynamic exclusion time of 20 s was applied.

### Database searching and data analysis

Proteins were searched against a concatenated target-reverse database MG8 (*Magnaporthe* comparative Sequencing Project, Broad Institute of Harvard and MIT), and identified using the Sequest HT algorithm in Proteome Discoverer 1.4 (Thermo Scientific, San Jose, CA). Search parameters used a 5 ppm precursor mass and 0.02 Da fragment mass tolerance and allowed up to 2 missed cleavage sites. False discovery rate calculations were generated using Percolator at a 1% protein false discovery rate (FDR). Peptide spectral matches (PSMs) were normalized across technical and biological replicates, and treatment conditions using the total spectral counts method as previously described [21]. Differential protein expression was calculated by dividing the normalized PSMs of the nitrogen-starved state by the nitrogen treated state. Statistical significance of unique protein identification and protein fold-changes between the treatments was determined using a pairwise Student’s t-test with a cutoff of *p* ≤ 0.05.

Functional annotation was performed using David Algorithm (version 6.7) [22]. Biological pathways of identified proteins were predicted by searching the *M. oryzae* KEGG pathway database (release 79) [23]. Subcelluar localizations to all predicted proteins of the *M. oryzae* version 8 genome were assigned by the WoLF PSORT program [24]. The 500 bp upstream of open reading frames were searched for HGATAR domains (H = A, C or T and R = A or G) using the POCO motif finding program [25] and the presence of HGATAR motifs were compared between gene of interests and background genes.

## Results and discussion

### Proteome identification in nitrogen supplemented and nitrogen depleted growth condition

**Fig. 1:**
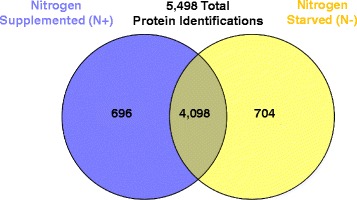
Number of proteins identified from *M. oryzae* in this study. Venn diagram shows the number of proteins identified in either N+ and N- only and those shared between both conditions

In this study, the protein profiles of *M. oryzae* 70–15 mycelia grown under nitrate supplemented or nitrogen depleted conditions were compared. The fungus was initially cultivated in nutrient rich complete media for 3 days and then switched to minimal media with or without nitrogen sources for an additional 12 h. To generate a detailed interrogation of protein changes associated with nitrogen starvation, highly sensitive, global MS/MS technologies for protein detection coupled to advanced annotation tools were employed. A total of eighteen injections from samples consisting of two nitrogen treatments (nitrate supplemented (N+) and nitrogen depleted (N-)), three biological replicates and three technical replicates were investigated. On average, 153,000 tandem mass spectra were collected corresponding to an average of 23,648 MS^1^ peptides per injection. Overall, the whole proteomic dataset mapped to a total of 5498 *M. oryzae* proteins (representing >42% of the 12,991 predicted proteins in *M. oryzae* V8 annotation proteome) with a 1% FDR (Fig. 1, Additional file 1: Table S1). There were 4098 proteins shared between the two treatments, 704 found only in N-, and 696 only in N+. In previous work, employing FASP and anion StageTip fractionation analyzed on an Orbitrap XL, 3200 proteins were identified in *M. oryzae* [26]. The method used in this study did not include fractionation, which resulted in more complex samples. Nevertheless, the employ of newer MS technologies with significant higher MS/MS scan rates resulted in an increase in proteome coverage by more than 75%. Similar results have been recently reported in yeast where it is now possible to identity nearly 4000 proteins representing ~60% of the theoretical proteome without sample fractionation [27]. This important advancement enabling the use of non-fractionated samples now paves the way for more sophisticated and complex proteome studies in the future.

**Fig. 2:**
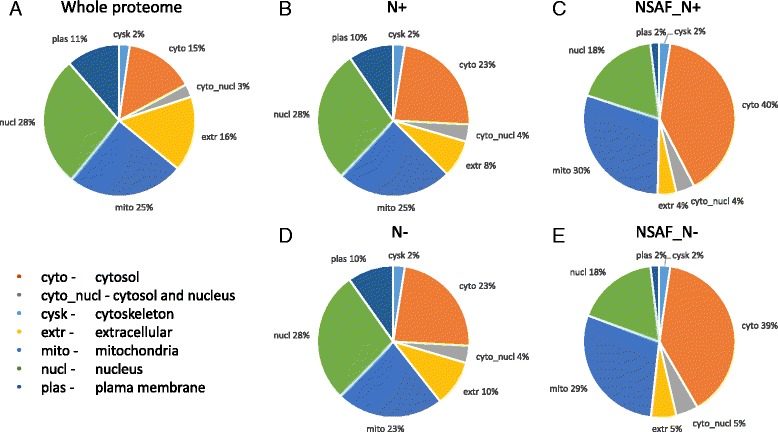
Subcellular distribution patterns of *M. oryzae* proteins. Subcellular localization of: **a**. 12,991 predicted *M. oryzae* proteins, **b**. 4794 identified proteins in nitrate supplemented condition, N+ and **c**. 4802 proteins in nitrogen starved condition, N-. **d**. and **e**. The relative protein abundance of proteins (NSAF_N+, NSAF_N-) as indicated by the percentage of the sum of NSAF values in each localization category

The cellular localization of *M. oryzae* proteins was analyzed using the prediction tool, WoLF PSORT [24]. The WoLF PSORT distribution of the theoretical *M. oryzae* database (12,991 proteins) localized 15%, 28%, 25%, and 16% of the proteins to cytosol, nucleus, mitochondria, and extracellular regions, respectively (Fig. 2a). By comparison, among the 4794 identified proteins in the N+ condition, 23% were cytosolic, 28% nuclear, 25% mitochondrial and 8% extracellular (Fig. 2b). Among the 4802 identified proteins in the N- condition, 23% were cytosolic, 28% nuclear, 23% mitochondrial and 10% extracellular (Fig. 2d). The N+ and N- proteome both showed a clear enrichment of cytosolic proteins and reduction of extracellular proteins.

Protein detection is likely to be directly related to the amount of protein present in the tissue and thus may reflect the general level of protein expression in the N+ and N- conditions (high for cytosolic proteins and low for extracellular proteins). To test this hypothesis, the expression level according to cellular localization was evaluated. The relative cellular protein expression was measured by calculating the normalized spectral abundance factor (NSAF) and summed for each subcellular localization category [28, 29].

This analysis showed that in both nitrogen supplemented and starved conditions, the cytoplasmic and mitochondrial proteins represented not only the largest groups of identified proteins but also embodied proteins of high abundance. In contrast, extracellular proteins were underrepresented among the identified proteins and generally contained proteins of lower abundance. However, we cannot exclude the possibility that some extracellular protein were lost during sample preparation. Although, we identified the nuclear and plasma membrane proteins in the same proportion as the whole proteome, these proteins showed relatively lower protein abundance (Fig. 2c, e). These results were very similar to our previous analysis of *M. oryzae* conidial proteome [30] and suggests the general enrichment of cytosolic and mitochondrial proteins in quantity and quality and underrepresentation of extracellular proteins in *M. oryzae* tissues.

**Fig. 3:**
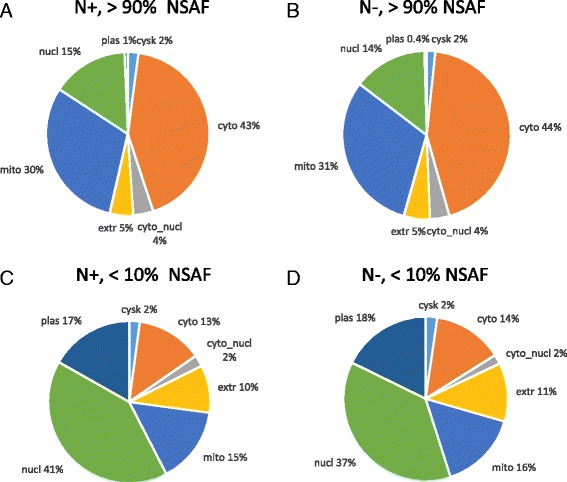
Distribution of cellular localization of the most and the least abundant proteins identified in *M. oryzae* under nitrate nitrogen (N+) and nitrogen starved (N-) conditions. Proteins ranking in the top 90% (>90%, **a** and **b**) and bottom 10% (<10%, **c** and **d**) in NSAF value were grouped respectively according to the expected cellular location. Localization categories are shown in Fig. 2

Follow-up studies were performed on the highest (≥ 90 percentile in NSAF value) and lowest abundant (≤ 10 percentile NSAF value) proteins. Among the most abundant proteins in the N+ group, the majority were cytosolic (43%) followed by mitochondrial (30%) proteins and 15% nuclear proteins (Fig. 3a). A similar distribution was observed for highly abundant proteins in the N- condition (Fig. 3b). The highly abundant cytosolic proteins in both N+ and N- were annotated as subunits of ribosome and proteasome or involved in glycolysis, amino acid biosynthesis, aminoacyl-tRNA biosynthesis, starch and sucrose metabolism or fatty acid biosynthesis. Based on KEGG pathway analysis, among the highly expressed mitochondrial proteins, 12 proteins, including NADH dehydrogenases, F-type ATPases, cytochrome c oxidase and cytochrome c reductase, were implicated in the processes related to oxidative phosphorylation. TCA cycle and amino acid biosynthesis related proteins and components of the ribosome were also identified in the mitochondria proteins (Additional file 1: Table S1). The most abundant extracellular proteins included cell wall modifying enzymes, such as cell wall glucanosyltransferase (MGG_00592), glucan 1,3-beta-glucosidase (MGG_04689) and beta-glucosidase 1 (MGG_09272) and different types of proteases, such as dipeptidyl-peptidase V (MGG_07877), subtilisin-like proteinase Spm1 (MGG_03670) and carboxypeptidase Y (MGG_05663). In sum, proteins associated with growth and metabolism were the most abundant.

Among the lowest abundant proteins, nuclear, plasma membrane and extracellular proteins were over-represented, whereas cytosolic and mitochondrial proteins were under-represented in both the N+ and N- (Fig. 3c, d).

### Proteome changes during nitrogen starvation

**Fig. 4:**
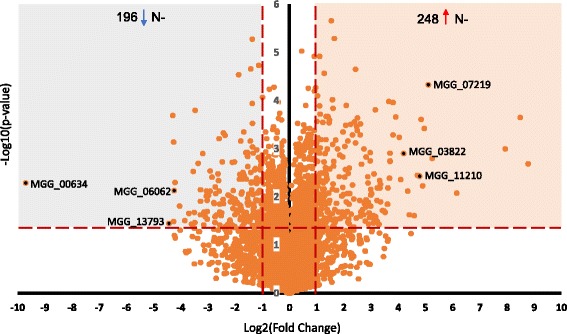
Volcano plot of *M. oryzae* proteins from nitrate nitrogen (N+) and nitrogen starved (N-) conditions. Out of 5498 proteins identified, 444 were differentially expressed. Two hundred and forty eight were induced and 196 repressed (≥2 fold-change, *p*-value ≤0.05). MGG_00634 (nitrite reductase), MGG_06062 (nitrate reductase), MGG_13793 (nitrate transporter), MGG_07219 (melanin biosynthesis polyketide synthase), MGG_03822 (peptidase family T4) and MGG_11210 (beta-glucosidase 1) are highlighted

Relative quantification of protein abundance between the N+ and N- conditions was determined using PSMs. One hundred fifteen and 70 proteins were found to be significantly unique (Student’s T test, *p* ≤ 0.05) in the N- and N+ groups, respectively. Among the 4098 overlapping proteins, 444 proteins were identified as significantly regulated, with a 2-fold or greater change between the groups (*p*-value <0.05). 248 proteins were over-expressed and 196 proteins were repressed in the N- group compared with N+ (Fig. 4). Combining the uniquely identified with the differentially expressed proteins resulted in 363 induced and 266 repressed proteins in fungal mycelia undergoing nitrogen starvation (Additional file 2: Table S2), which represented 11% of the total proteins identified. To investigate the potential biological implications of these 629 differentially expressed proteins, David GO Functional analysis v6.7 [22] were applied (Table 1). Groups of proteins for melanin biosynthesis, amino acid transport, cell morphogenesis, carboxylic acid transport, ion transport and tyrosine metabolic process were enriched during nitrogen starvation. By contrast, proteins for protein synthesis, nitrate assimilation, carbohydrate biosynthetic process port and porphyrin biosynthesis (Table 1) were repressed. In-depth discussion of these proteins is provided below to gain further insight into the relationship between nitrogen starvation and fungal development, signaling and effector protein expression.

**Table 1:** Enriched functional groups among differentially expressed proteins during nitrogen starvation

Expression	GO ID	GO Description	Protein ID	pValue	Enrichment Score
ID	GO:0042438	melanin biosynthetic process	MGG_07216, MGG_07219, MGG_02252, MGG_05059	0.00	23.8
ID	GO:0046942	carboxylic acid transport	MGG_05128, MGG_00289, MGG_14115, MGG_01054, MGG_07606, MGG_11327	0.02	4.0
ID	GO:0000902	cell morphogenesis	MGG_04703, MGG_06033, MGG_03703, MGG_02781	0.00	13.2
ID	GO:0006570	tyrosine metabolic process	MGG_02252, MGG_06691, MGG_05059	0.02	12.7
ID	GO:0006811	ion transport	MGG_04159, MGG_03299, MGG_10027, MGG_06118, MGG_01054, MGG_02124, MGG_09119, MGG_04135, MGG_10634, MGG_05281, MGG_09063	0.04	2.0
ID	GO:0006865	amino acid transport	MGG_05128, MGG_00289, MGG_14115, MGG_07606, MGG_11327	0.05	3.5
RP	GO:0006412	translation	MGG_13783, MGG_01165, MGG_00161, MGG_07154, MGG_06935, MGG_08323, MGG_04042, MGG_02511, MGG_05031, MGG_04455, MGG_10825, MGG_06468, MGG_05275, MGG_09301, MGG_06744, MGG_14349, MGG_05647	0.00	2.3
RP	GO:0042128	nitrate assimilation	MGG_00634, MGG_06062, MGG_04144	0.01	18.0
RP	GO:0042401	biogenic amine biosynthetic process	MGG_10533, MGG_11574, MGG_07454	0.02	12.0
RP	GO:0008610	lipid biosynthetic process	MGG_06288, MGG_03343, MGG_08474, MGG_13185, MGG_09239, MGG_06935, MGG_06133, MGG_07543, MGG_00806	0.03	2.5
RP	GO:0006418	tRNA aminoacylation for protein translation	MGG_13783, MGG_00161, MGG_01165, MGG_05275, MGG_04042	0.04	3.9
RP	GO:0034637	cellular carbohydrate biosynthetic process	MGG_00865, MGG_13185, MGG_06935, MGG_00450	0.04	5.1
RP	GO:0006779	porphyrin biosynthetic process	MGG_04860, MGG_06446, MGG_10321	0.05	8.3

*Functional annotation clustering analysis was performed with 629 differentially expressed proteins using David GO v 6.7. Groups with enrichment score ≥ 2 and p-value ≤0.05 are presented for the induced (ID) and repressed (RP) proteins during nitrogen starvation

### Redirection of nitrogen metabolism pathway

Fungi have evolved mechanisms to uptake and convert inorganic nitrogen compounds from the environment to organic nitrogen compounds, which are incorporated into cellular substances including amino acids [31]. By so-called nitrogen assimilation, extracellular nitrate is transported into the cell by nitrate transporters and reduced to nitrite by nitrate reductase. This is further reduced to ammonia by nitrite reductase [31]. Our data suggests that in *M. oryzae*, this nitrogen assimilation process is highly regulated by controlling the production of the key enzymes. During nitrogen starvation, the production of nitrate reductase (MGG_06062) and nitrite reductase (MGG_00634) were significantly reduced, both by more than 100 fold. Further, levels of the nitrate transporter (MGG_13793) were significantly reduced in N- by more than 20 fold. Thus when nitrate nitrogen is limiting in the environment, fungal resources are likely directed elsewhere and enzymes required for nitrogen assimilation are greatly reduced.

Elevated levels of nitrogen regulators such as NUT1 (MGG_02755) were also observed under nitrogen starvation. NUT1 is a member of GATA family transcription factors, which includes AreA from *A. nidulans* and Nit2 from *N. crassa*, which are well known global nitrogen regulators in nitrogen catabolite repression (NCR) [5, 32]. Typically, these transcription factors activate expression of nitrogen metabolic genes under nitrogen starvation. They contain Cys2/Cys2 type zinc fingers which recognize the consensus DNA sequences HGATAR in promoter sequences of target genes. Interestingly, although NUT1 is highly upreguated by nitrogen starvation in our studies, proteins involved in assimilation of nitrate, which are typically regulated by NUT1, were not up-regulated as noted above. There are possible explanations for this apparent contradictory observation. In *Aspergillus nidulans*, for example, the activity of AreA is subject to co-repression by NmrA [33]. *M. oryzae* contains 3 homologs of NmrA, two of which Nmr1 and Nmr3 interact with NUT1 [34]. Also, other proteins such as the highly conserved AreB, have been shown to act as regulators of nitrogen metabolic genes [35]. Thus, it is possible that when nitrogen is lacking co-repressors or other proteins regulate the action of NUT1. In addition, other unknown factors may affect the translation or stability of nitrate assimilation proteins.

NUT1 plays a role in virulence of *M. oryzae*. Nut1 null mutants cause reduced lesion numbers compared to the wild-type [7]. NUT1 is also required for the growth on several non-preferred secondary nitrogen sources and regulates gene expression of the hydrophobin like effector, MPG1 under nitrogen starvation [7, 36]. GATA transcription factors from other plant pathogenic fungi have been shown to be key for pathogenesis. For example, CLNR1 mutants of *Colletotrichum lindemuthianum* are non-pathogenic [8]. Like in *M. oryzae*, these mutants can produce appressoria, but invassive growth is hampered. Reduced virulence was also reported in the FNR1 disrupted mutant in *Fusarium oxysporum* f. sp. *lycopersici* [37].

Plants contain a number of nitrogen sources that may be used by pathogenic fungi. For example, γ-aminobutyric acid (GABA) is a major metabolite in apoplast of tomato and other plants. During infection, GABA levels have been shown to rise [38, 39]. Fungi uptake GABA via GABA permease. In *A. nidulans*, expression of GABA permease, GabA is under NCR and regulated by the GATA transcription factor AreA. Here, we observed protein expression of a GABA permease MGG_14115 in *M. oryzae* increased more than three-fold during nitrogen starvation. However, there is no direct evidence that GABA permease is regulated by NUT1. In our previous work, gene expression of both GABA permease and NUT1 were found to be increased by nitrogen starvation [13]. This strongly suggests that in *M. oryzae*, nitrogen limitation triggers both gene expression and accumulation of proteins of major players for nitrogen scavenging including uptake of available nitrogen sources, potentially including those encountered during infection.

Our data also showed co-occurrence between nitrate metabolism and sulfate metabolism during nitrogen starvation. Key enzymes for sulfate assimilation including phosphoadenosine phosphosulfate reductase (MGG_03662), sulfite reductase flavoprotein component (MGG_00929), sulfite reductase (MGG_04144), sulfate adenylyltransferase (MGG_15027) were significantly reduced during nitrogen starvation. It has been reported in other biological systems that sulfate assimilation is regulated by availability of nitrogen sources and nitrogen starvation represses gene expression and enzyme activity of proteins involved in sulfate assimilation [40–42]. This may be a result of general repression of pathways involved in protein synthesis preventing the accumulation of high levels of sulfur containing amino acids such as cysteine and methionine [43].

### Melanin biosynthetic process

Availability of nitrogen regulates the synthesis of broad range of secondary metabolites in fungi [44]. Melanin is one of the most thoroughly studied secondary metabolites in fungi including pathogenic fungi. Most fungi produce DHN melanins using 1,8-dihydroxynaphthalene (DHN) as precursor. Highly conserved in pathogenic filamentous fungi, DHN melanin is synthesized via the polyketide pathway. Proteins involved in this process include polyketide synthase (PKS), 1,3,6,8-tetrahydroxy-naphthalene reductase (4HNR), trihydroxy-naphthalene reductase (3HNR) and scytalone dehydratase (SCD). In *M. orzyae*, melanin biosynthesis is known to be essential for infection of the host plant [45]. A highly melanized outer cell wall of the appressorium facilitates high turgor pressure that is essential for successful penetration into the host plant [46]. Genes involved in melanin synthesis including PKS (MGG_07219), 3HNR (MGG_07216), 4HNR (MGG_02252) and SCD (MGG_05059) have been functionally characterized and shown to be essential for fungal development and pathogenicity [45]. For example, gene deletion of PKS (MGG_07219) resulted in the production of non-functional appressoria, which failed to infect the host plant [15, 47].

In a previous study, we showed that genes involved in this pathway were highly induced during appressorium formation and were under cAMP signaling pathways in *M. orzyae* [15]. Here, we found that proteins involved in melanin biosynthesis increased in response to nitrogen starvation in *M. oryzae*. All the principal enzymes in this pathway, PKS (MGG_07219), 3HNR (MGG_07216), 4HNR (MGG_02252) and SCD (MGG_05059), were significantly induced during nitrogen starvation. In addition, we observed mycelia were more pigmented under nitrogen starvation compared to the non-starved condition, which is likely the result of increased melanin production at least in part (Additional file 3: Figure S1). PKS (MGG_07219), 4HNR (MGG_02252) and SCD (MGG_05059) genes contains the consensus DNA sequences HGATAR in their promoter region (Additional file 2: Table S2), which suggests that expression of the melanin biosynthesis genes may be controlled by a GATA transcription factor under nitrogen starvation, possibly by NUT1.

In other fungal pathosystems, increased melanin biosynthesis has been suggested to enable infection. For example, based on the transcriptomic profiling, increased melanin biosynthesis during nitrogen starvation was proposed to be important for pathogenesis by the wilt pathogen, *V. dahliae* [14]. Increased melanin may also confer other beneficial properties. Increased melanin biosynthesis may provide protection of fungal cells from ROS during infection. For example in *Colletotrichum acutatum*, ROS accumulation has been reported during nitrogen limiting conditions [48]. With a strong affinity for metals, melanin acts as very effective scavenger of those free radicals. DHN melanin also protects fungal cells against permanganate, hypochlorite and neutrophil oxidative burst [49, 50]. Melanin extracted from the medical fungus, *Auricularia auricular,* exhibited strong radical scavenging activities [51]. Thus, for pathogenic fungi, melanin biosynthesis is crucial for both the infection process and protection against the oxidative burst associated with host defense responses.

In other studies, expression of genes in a number of secondary metabolite gene clusters, which include polyketide synthase, have been shown to be dependent on the quantity and quality of the nitrogen source. For example, in *Fusarium fujikuroi* among 20 PKS gene clusters, the expression of 13 was influenced by nitrogen source [52]. In our experiments, with the exception of the PKS involved in melanin biosynthesis (MGG_07219), the expression of only one other PKS (MGG_15100) was affected by nitrogen source.

### Increased activity of protein degradation

The largest group of proteins induced during nitrogen starvation was proteins involved in recycling of nitrogen sources. Exopeptidases (MGG_03822, MGG_07704, MGG_07981, MGG_09530) and metallopeptidases (MGG_01970, MGG_06643, MGG_07704, MGG_07981, MGG_09530) were induced as were 7 different endopeptidases (MGG_00922, MGG_02514, MGG_02849, MGG_02898, MGG_04031, MGG_06643, MGG_11021). It is noteworthy that certain metallopeptidases, such as AVR-PITA in *M. oryzae*, have roles associated with virulence [53]. Once proteins are degraded into amino acids, they must be translocated quickly through amino acid transporters. Five amino acid transporters (MGG_05128, MGG_00289, MGG_14115, MGG_07606 and MGG_11327) were enriched under nitrogen starvation. Increased synthesis of protein degrading enzymes and amino acid transporters suggests that during nitrogen starvation, fungi aggressively and efficiently recycle/scavenge available nitrogen sources from their own cells and/or surrounding resources.

To identify nitrogen metabolism proteins which are potentially regulated by GATA transcription factors, HGATAR motifs within the promoters of identified proteins were searched using the POCO motif finding program [25]. The occurrence and frequency of this motif, according to the protein expression pattern, was compared in regard to nitrogen starvation (Additional file 4: Table S3). Among 5498 *M. oryzae* proteins we identified, genes encoding 3831 proteins (69.7%) have HGATAR motifs in their promoter region. Most proteins had single (48.2%) or double (32.2%) motifs. A similar percentage of proteins containing the motif was found across the subset of induced and repressed proteins. However, promoters with high numbers of HGATAR motifs were enriched in induced proteins. For induced proteins, 7.6% contained 4 or more HGATAR motifs per promoter compared to 5.8% in repressed or not differentially expressed proteins. Surprising, these induced proteins with high numbers of HGATAR motifs had similar biological function; protein degradation and amino acid modification. These proteins contained an amino acid transferase (MGG_09919), an amidohydrolase (MGG_10507), an L-asparaginase 1 (MGG_04119), a dipeptidase (MGG_09530), a urease (MGG_01324) and a L-serine dehydratase (MGG_06950). These findings suggests that protein catabolism during nitrogen starvation is tightly controlled by GATA transcription factors, likely including NUT1 in *M. orzyae*.

### Increased production of putative extracellular proteins during nitrogen starvation

**Fig. 5:**
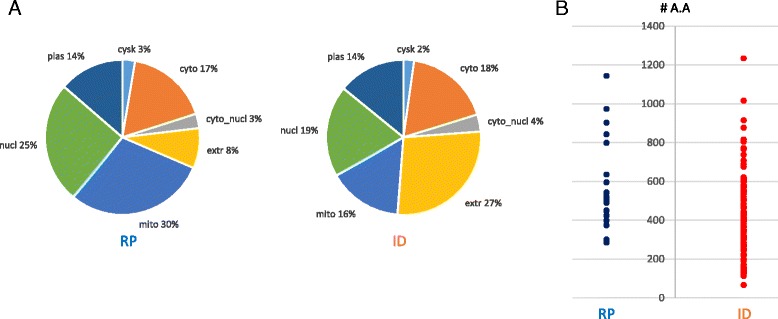
Features of proteins differentially repressed (RP) and induced (ID) by nitrogen starvation. **a**. Subcellular localization and **b**. Size distribution of extracellular proteins

Among the proteins enriched during nitrogen starvation, 27% of the proteins (99 proteins out of 363 proteins) were predicted to be extracellular proteins in contrast to only 8% of proteins (22 proteins out of 266 proteins) among repressed proteins. On the other hand, nuclear and mitochondrial proteins were more represented among repressed proteins (Fig. 5a). In plant pathogenic fungi, a number of small secreted proteins have been shown to act as effector proteins enabling pathogen virulence and inplanta growth [54, 55]. Interestingly, all 22 small secreted proteins (< 250 a.a) were identified exclusively among proteins induced by nitrogen starvation (Fig. 5b). Most of these proteins are unknown hypothetical proteins without any functional domains or orthologs in other organisms (Table 2).

**Table 2:** List of small extracellular proteins enriched during nitrogen starvation

Protein ID	Annotation	# A.A	PSM_N+	PSM_N-	FC	*p* value	# HGATAR	Cys > 3%
MGG_09842	hypothetical protein	171	0	521.8	–	0.00	0	Y
MGG_13009	hypothetical protein	226	0	250.4	–	0.00	0	
MGG_00052	hypothetical protein	225	0	231.7	–	0.00	1	
MGG_05344	SnodProt1	138	56	120.7	2.15	0.00	0	
MGG_04323	hypothetical protein	244	46.2	97.1	2.10	0.04	1	
MGG_07571	LysM domain-containing protein	143	0	64.9	–	0.00	3	Y
MGG_00081	hypothetical protein	190	0	51.3	–	0.01	1	
MGG_07850	hypothetical protein	245	13.4	50.7	3.78	0.01	3	
MGG_07782	dehydroquinase class II	158	10.4	46.3	4.45	0.00	3	
MGG_03791	hypothetical protein	130	0	41.1	–	0.02	0	Y
MGG_13654	hypothetical protein	124	0	38.1	–	0.00	1	Y
MGG_06234	hypothetical protein	142	0	34	–	0.01	2	Y
MGG_06359	hypothetical protein	248	0	28.9	–	0.01	0	
MGG_15022	hypothetical protein	143	0	28.8	–	0.02	1	
MGG_12247	hypothetical protein	221	0	20.6	–	0.01	0	
MGG_10456	hypothetical protein	150	0	14.4	–	0.00	0	Y
MGG_10315	hydrophobin-like protein MPG1	113	2.1	13.4	6.47	0.02	0	Y
MGG_03442	hypothetical protein	246	4.2	12.4	2.96	0.02	2	
MGG_07246	hypothetical protein	200	0	9.3	–	0.00	1	Y
MGG_07791	surface protein 1	135	0	9.2	–	0.03	0	Y
MGG_02085	FAD-linked sulfhydryl oxidase ALR	218	0	8.3	–	0.02	2	
MGG_10604	hypothetical protein	66	0	5.2	–	0.04	1	

*Number of spectral counts in nitrogen starved (PSM_N-) and nitrate nitrogen supplemented (PSM_N+), fold change (FC) and p-value are presented. The number of HGATAR domains in the promoter region and cysteine content of the protein are also shown

Among the small proteins, the hydrophobin effector MPG1 (MGG_10315) was significantly (>6 fold) induced during nitrogen starvation. MPG1 is a small hydrophobic protein involved in the surface interaction during plant infection. The gene is highly expressed in fungi during plant infection and when starved for nitrogen and carbon sources. Mpg1 mutants are non-pathogenic [2, 56]. In addition, MPG1 is regulated by NUT1 under nitrogen starvation conditions [36, 57]. Other effector genes, such as Avr9 from *Cladosporium fulvum*, are highly expressed during infection and under nitrogen starvation conditions. Interestingly, the promoter of Avr9 contains mutiple GATA binding sites that are necessary for induction by the GATA transcription factor NRF1. NRF1 is also required for virulence by *C. fulvum* [1, 2].

We also found MGG_05344, Snodprot1, to be significantly induced by nitrogen starvation. Snodprot1 is associated with pathogenicity and was first identified to be expressed during plant infection by the wheat blotch pathogen *Parastagonospora nodorum* [58]. Application of the Snodprot1 protein from *M. oryzae* elicited host defense responses in rice and induced host cell death [59]. The protein was found to be essential for rice infection by *M. orzyae* [60].

*M. oryzae* has several lysin motif (LysM) containing proteins and one of them, MGG_07571, was expressed only in nitrogen starved condition in this study. Other LysM containing secreted proteins such as ECP6 in *Cladosporium fulvum* and SLP1 in *M. oryzae* are well characterized effector proteins. LysM domains bind chitin and prevent chitin triggered immune responses in the host plant [61, 62].

MGG_07791, a secreted protein homologous to a major cell surface protein CLSP1 in the bean pathogen *C. lindemuthianum*, was also induced by nitrogen starvation. This protein may be involved in adhesion of the pathogen to the host. However, the gene is conserved in other filamentous fungi including *F. graminearum* and *A. nidulans* [63].

Nine of the 22 small secreted proteins, including MPG1, Surface protein1 (MGG_07791), and LysM protein (MGG_07571) have more than 3% cysteine content (Table 2). High cysteine content would likely confer structural stability to the secreted protein that in a hostile external environment such as at the host interface may enable them to work as effector proteins [64].

### Reduction of de novo protein production

De novo protein synthesis is a very energy consuming process. The largest functional group (17 of 266) of repressed proteins was associated with translation. These included five tRNA synthetases (MGG_01165, isoleucyl-tRNA synthetase; MGG_00161, lysyl-tRNA synthetase; MGG_04042, leucyl-tRNA synthetase; MGG_05275, glutamyl-tRNA synthetase; and MGG_13783, aspartyl-tRNA synthetase) and MGG_01021, a tRNA (guanine-N(7)-)-methyltransferase. Protein synthesis is mediated by the ribosome complex, which is composed of dozens of proteins in both the large and small units. In our study, a number of structural constituents of the large subunit (MGG_02511, MGG_04455, MGG_05647, MGG_06468, MGG_10825) and small subunit (MGG_06744, MGG_06935) as well as mitochondrial ribosomal units (MGG_08323, MGG_09301) were repressed during nitrogen starvation. MGG_14349, a translation release factor was also down regulated.

Under nitrogen limiting conditions, active growth becomes severely challenging. Thus, an overall down-turn in the production of proteins involved in protein synthesis machinery would be not unexpected. Instead, efforts are directed to processes that can help fungi survive nitrogen limitation, including scavenging and in the case of pathogens exploiting host resources.

## Conclusion

Technological improvements in tandem mass spectroscopy and data analysis has enabled a thorough investigation of proteome changes when the rice blast fungus encounters nitrogen starvation. Representing more than 40% of the entire proteome, 5498 proteins were identified during mycelial growth with/without nitrogen sources employing total unfractionated protein samples. In depth analysis of 629 differentially enriched proteins afforded new insight into fungal responses to nitrogen starvation. Proteins associated with melanin accumulation and nitrogen scavenging were observed to increase under nitrogen stress, whereas protein synthesis and proteins associated with nitrogen assimilation decreased. This study further uncovered that nitrogen limitation triggers accumulation of secreted proteins, which may enable host plant infection or function as effector proteins. We expect further functional characterization of those differentially expressed proteins will help broaden the scope of future studies.

## Additional files

Additional file 1: Table S1. Protein Identification. (XLSX 316 kb)

Additional file 2: Table S2. List of differentially expressed proteins. (XLSX 66 kb)

Additional file 3: Figure S1. Increased pigmentation during nitrogen starvation. (PPTX 161 kb)

Additional file 4: Table S3. Distribution of HGATAR motifs in *M. oryzae* gene promoters. (XLSX 11 kb)

## Abbreviations

3HNR: trihydroxy-naphthalene reductase
4HNR: tetrahydroxy-naphthalene reductase
A.A.: Amino acid
ACN: Acetonitrile
AGC: Automatic gain control
Cyclic AMP: Cyclic adenosine monophosphate
DHN: Dihydroxynaphthalene
DTT: Dithiothreitol
EDTA: Ethylenediaminetetraacetic acid
FA: Formic acid
FASP: Filter aided sample preparation
FDR: False discovery rate
GABA: Gamma-aminobutyric acid
HPLC: High performance liquid chromatography
KEGG: Kyoto Encyclopedia of Genes and Genomes
LC: Liquid chromatography mass spectrometry
LC-MS/MS: Liquid chromatography-tandem mass spectrometry
LysM: Lysin motif
MS: Mass spectrometry
NCR: Nitrogen catabolic repression
NEM: N-ethylmaleimide
NSAF: Normalized spectral abundance factor
PSM: Peptide spectral match
ROS: Reactive oxygen species
SCD: Scytalone dehydratase
TCA cycle: Tricarboxylic acid cycle
