# Supplementary material for: Comparative proteomic analysis between nitrogen supplemented and starved conditions in Magnaporthe oryzae
Source: Proteome Sci. 2017 Nov 13;15:20. doi: 10.1186/s12953-017-0128-y (PMC5684745; doi:10.1186/s12953-017-0128-y)
Supplement: Supplementary file 3 — Increased pigmentation during nitrogen starvation. (PPTX 161 kb) [file 12953_2017_128_MOESM3_ESM.pptx]

## Slide 1
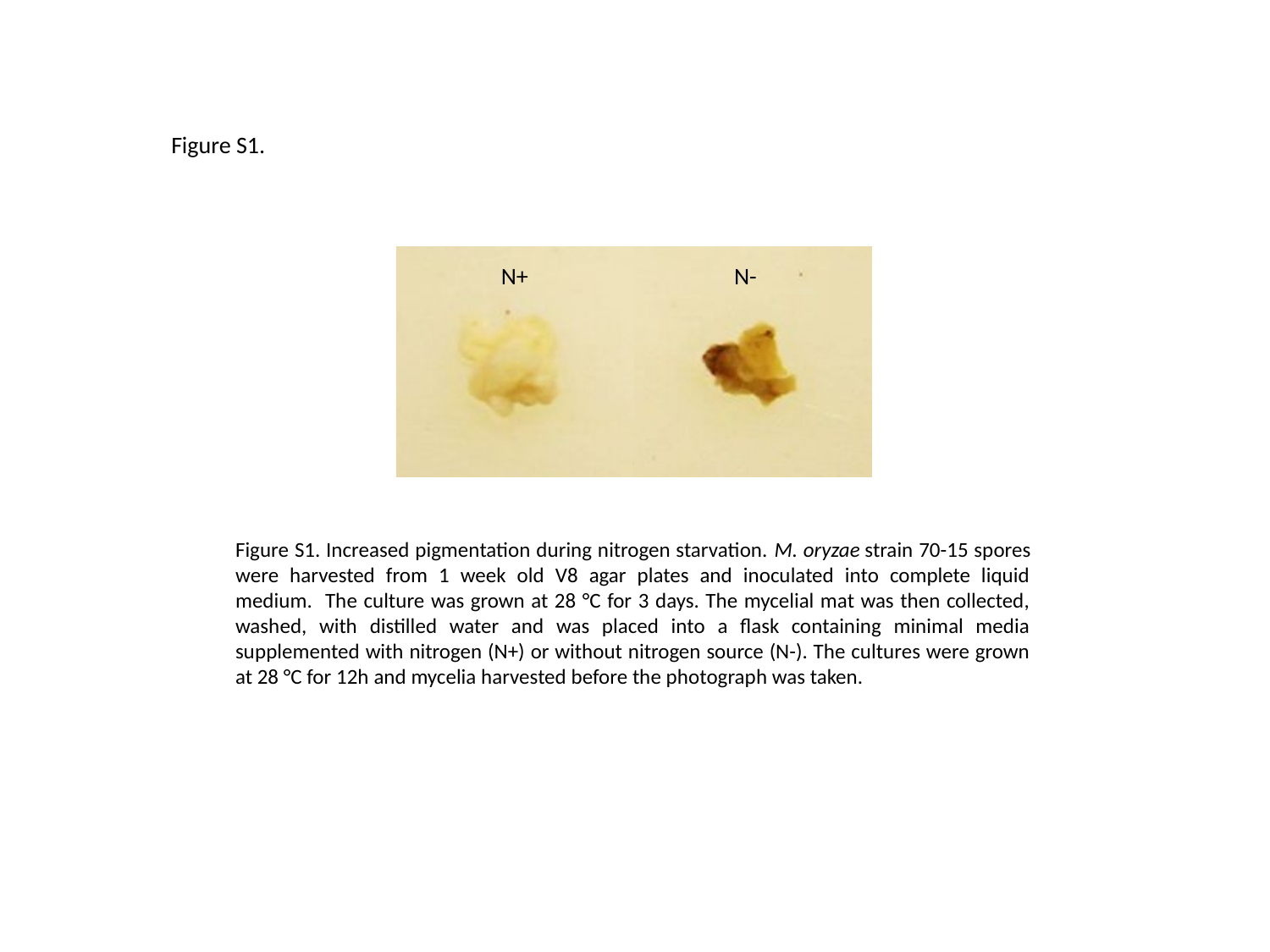

Figure S1.
 N+ N-
Figure S1. Increased pigmentation during nitrogen starvation. M. oryzae strain 70-15 spores were harvested from 1 week old V8 agar plates and inoculated into complete liquid medium. The culture was grown at 28 °C for 3 days. The mycelial mat was then collected, washed, with distilled water and was placed into a flask containing minimal media supplemented with nitrogen (N+) or without nitrogen source (N-). The cultures were grown at 28 °C for 12h and mycelia harvested before the photograph was taken.
